# Fabrication and properties of acellular porcine anulus fibrosus for tissue engineering in spine surgery

**DOI:** 10.1186/s13018-014-0118-z

**Published:** 2014-12-03

**Authors:** Lien-Chen Wu, Chang-Jung Chiang, Zen-Hao Liu, Yang-Hwei Tsuang, Jui-Sheng Sun, Yi-You Huang

**Affiliations:** Institute of Biomedical Engineering, College of Engineering, College of Medicine, National Taiwan University, No.1, Sec.1, Jen-Ai Road, Taipei, Taiwan; Department of Orthopedics, Shuang Ho Hospital, Taipei Medical University, Taipei, Taiwan; Institute of Clinical Medicine, National Yang-Ming University, Taipei, Taiwan; Department of Orthopaedics, National Taiwan University Hospital HsinChu Branch, HsinChu, Taiwan

**Keywords:** Anulus fibrosus, Decellularization, Acellular, Disc degeneration, Intervertebral disc, Tissue engineering

## Abstract

**Background:**

Over the last few years, new treatments for a damaged intervertebral disc (IVD) have included strategies to repair, replace, or regenerate the degenerative disc. However, these techniques are likely to have limited success, due to insufficiently effective means to address the damaged anulus fibrosus (AF). Here, we try to develop a bioprocess method for decellularization of the xenogeneic AF tissue, with a view to developing a scaffold as a potential candidate for clinical application in spinal surgery.

**Methods:**

Porcine AFs were decellularized using freeze-thaw cycles, followed by various combined treatments with 0.1% sodium dodecyl sulfate (SDS) and nucleases.

**Results:**

Hematoxylin and eosin (H & E) staining showed that decellularization was achieved through the decellularization protocols. Biochemical analyses revealed 86% reduction in DNA, but only 15.9% reduction in glycosaminoglycan (GAG) content, with no significant difference in the hydroxyproline content. There was no appreciable cytotoxicity of the acellular AF. Biomechanical testing of the acellular AF found no significant decline in stiffness or Young’s modulus.

**Conclusions:**

Porcine AF tissues were effectively decellularized with the preservation of biologic composition and mechanical properties. These results demonstrate that acellular AF scaffolds would be a potential candidate for clinical application in spinal surgery.

## Introduction

As the global population ages, degenerative disc disease (DDD) and lower back pain affect millions of people worldwide. Chronic back pain can manifest in relation to several clinical conditions, including disc herniation, radiculopathy, myelopathy, spinal stenosis, and instability. There is significant evidence of links between DDD and lower back pain although the pathogenesis of DDD has yet to be fully delineated.

Although degeneration of the intervertebral disc (IVD) is associated with the majority of cases of lower back pain, current treatment options are palliative rather than curative. Although the prevalent surgical procedures can be successful in relieving back pain in the short term, but they do not repair the disc nor restore the normal biological and mechanical properties of the human spine. Such procedures limit mobility or may otherwise further alter the biomechanics of the spine, leading to further degeneration of adjacent segments.

Discectomy is the most common surgical treatment of lumbar disc herniation. However, subsequent disc degeneration and recurrent disc herniation are major problems after surgery. To deal with DDD and damage of the anulus during discectomy, anulus fibrosus (AF) repair and reinforcement of the damaged AF, in addition to spinal surgery, are desired. Although there are commercially available implants for closing a damaged AF (the Inclose©, the Xclose©, and the Barricaid©), these techniques cannot prevent AF degeneration or maintain the biological AF structure in the long term [1,2]. AF closure is also essential for nucleus pulposus (NP) replacement strategies to prevent the NP replacement from leaking out from the disc.

Because of the limited intrinsic healing capacity and low cellularity of the AF, it is susceptible to degeneration and poor repair, similarly to articular cartilage [2,3]. Therefore, tissue engineering would be an ideal candidate for repair or substitution of degenerated discs with appropriate analogs. Several different types of biomaterials have been explored as possible candidates for AF repair, but none mimic the mechanical properties and compositional structure of AF [4,5]. An alternative strategy for developing a biomaterial with tissue engineering is to decellularize xenogeneic AF tissues by removing the immunogenic cells. The objective of decellularization is to remove all cellular and nuclear material while minimizing any adverse effect on the mechanical integrity, biological activity, and composition of the remaining scaffold. This method has been successfully utilized in various fields of tissue engineering, including cartilage, the meniscus, ligaments, and tendons [6,7].

Over the last few years, advanced development in the field of tissue engineering has resulted in various potential new strategies to repair, replace, or regenerate the degenerative disc [8-10]. Despite major advances, none has yet succeeded in clinical therapy [4].

In an attempt to preserve the integrity of the AF and to minimize damage to biomechanical functions of the disc, anular repair seemed to be the easiest method to achieve this goal [5]. Previously, we have described a new anular repair after discectomy in IVD degeneration [11]. While direct repair after discectomy can be expected to reduce the risk of recurrent disc herniation and has been shown to result in improved anular healing, the development of new therapeutic strategies to augment damaged AF tissues may offer new hope for alleviating chronic low back pain when direct repair is not possible [12]. The present study aims to develop a bioprocess method for decellularization of the xenogeneic AF tissue and proceeds to characterize the biochemical and biomechanical properties of a natural and acellular AF scaffold. The extent of decellularization was determined using histological, biochemical, and immune-histochemical techniques. The biocompatibility and biomechanical properties of the decellularized scaffold were also investigated to establish a potential protocol for clinical application.

## Materials and methods

### Tissue harvest

Fresh porcine lumbar spines were obtained *en bloc* from a local *abattoir* (Taoyuan County, Taiwan) within 2 h postmortem. The AF samples were harvested from the IVD by gently excising and washing in phosphate-buffered saline (PBS) to remove excess blood. Samples were then placed in neutral-buffered formalin for histological analysis or frozen and stored on a PBS moistened filter paper at −20°C.

### Decellularization methods

Decellularization was based on the methods developed by Booth et al. and modified by Stapleton [13,14]. Briefly, the AF samples were decellularized by exposing the tissue to five dry freeze-thaw cycles. The freezing step was at −80°C for 22 h, followed by thawing in an incubator at 37°C for 2 h. Samples were then incubated in a hypotonic buffer (10 mM Tris–HCl, pH 8.0), first at 4°C for 24 h, then at 37°C for 24 h, then in 0.1% sodium dodecyl sulfate (SDS; Sigma-Aldrich) at 45°C for 48 h, with agitation in the presence of protease inhibitors (aprotinin 10 KIU/mL; Sigma-Aldrich). After washing, the samples were incubated in DNase (50 U/mL; Sigma-Aldrich) and RNase (1 U/mL; Sigma-Aldrich) in a buffer (50 mM Tris–HCl, 10 mM magnesium chloride, and 50 mg/mL bovine serum albumin (BSA) at pH 7.5) for 3 h at 37°C with gentle agitation. Tissues were then washed in PBS at 37°C for 8 h three times.

### Histology

Tissue specimens were fixed in neutral-buffered formalin for 48 h, then embedded in paraffin wax and sectioned at 6-μm thickness with a microtome. Samples were characterized histologically using hematoxylin and eosin (H & E, Bios Europe, Skelmersdale, United Kingdom) staining to assay cellular content, sirius red staining to visualize collagen distribution, and alcian blue staining to localize glycosaminoglycan (GAG) content, as described by Stapleton et al. [14].

### Scanning electron microscopy (SEM)

Fresh-frozen AF and the decellularized AF scaffolds were harvested and fixed in 4% paraformaldehyde (wt./vol.) for 2 days. The samples underwent sequential dehydration and were sputtercoated with gold. Specimens were then evaluated and imaged using a SEM (JSM 5600, JEOL Ltd., Japan) to evaluate the ultrastructure of the AF surfaces.

### Determination of proteoglycan, collagen, and DNA content

#### Sulfated glycosaminoglycan assay

The proteoglycan content of the tissue was determined by measuring the amount of sulfated glycosaminoglycans in the papain-digested tissue using the 1,9-dimethylmethylene blue (DMMB; Sigma-Aldrich) dye binding assay and spectrophotometry [15]. AF tissue specimens were first lyophilized (*n* = 6, dry weight = 50 mg) and then digested in papain buffer (250 μL papain in PBS at pH 6.0 with 150 mM sodium chloride, 55 nM sodium citrate, 5 mM cysteine-HCl, and 5 mM Na_2_EDTA) at 60°C for 24 h. The supernatant fluid was measured at 530 nm using chondroitin sulfate as a standard.

#### Hydroxyproline assay

Collagen content was quantified using a commercially available assay kit (Hydroxyproline assay kit, BioVision, USA) [16]. AF specimens (*n* = 6) were first lyophilized and then hydrolyzed in 6 M hydrochloric acid (HCl) at 120°C for 3 h and neutralized using sodium hydroxide (NaOH). The hydroxyproline content was determined using the chloramine-T reagent assay and read using spectrophotometry at 560 nm. The concentration of hydroxyproline was then calculated by interpolation from a hydroxyproline standard curve.

#### DNA assay

The residual cells were determined by DNA assay (ReliaPrep™ gDNA Tissue Miniprep System, Promega, USA) [17]. DNA was extracted from both fresh and decellularized AF. Six AF specimens (wet weight = 25 mg) were used. DNA was extracted using a DNA isolation kit and then quantified according to the standard protocols by measuring the absorbance at 260/280 nm in a spectrophotometer (NanoDrop ND 1000, Thermo Fisher Scientific, United Kingdom).

### Cytotoxicity of decellularized AF in transwell insert model

Samples of decellularized AF were prepared and placed at the bottom of transwell insert wells before cytotoxicity testing. The transwells used in this study were 24 mm in diameter, with a membrane pore size of 0.4 μm (Costar, Transwell, Corning, NY, USA). The transwells were placed onto 24-well culture plates, then seeded with NIH3T3 fibroblasts (2 × 10^3^ cell/well) and incubated for 24, 48, and 72 h. Five wells per sample were prepared. In controls, cells were cultured in 24-well plates with transwell inserts but without any sample specimen. Following co-culture, the insert wells were removed. The viability of the target NIH3T3 fibroblasts was estimated by the 3-(4,5-dimethylthiazol-2-yl)-5-(3-carboxymethoxyphenyl)-2-(4-sulfophenyl)-2H-tetrazolium (MTS, Promega, USA) assay at 490 nm [18].

### Tensile test

**Figure 1:**
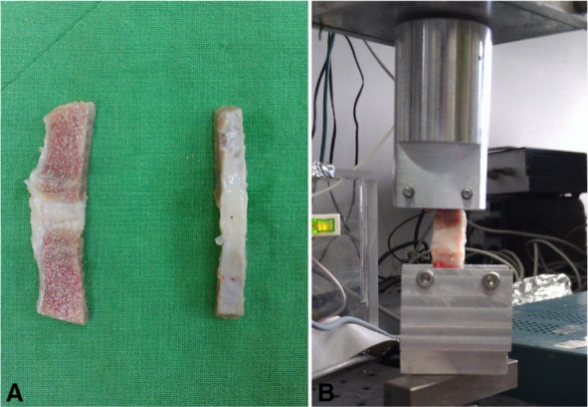
**Parallel bone-AF-bone specimens. (A)** Two parallel bone-AF-bone specimens were obtained from the same level disc and adjacent vertebrae. **(B)** Bone-AF-bone biomechanics were assessed using a materials testing system.

Due to the anatomy and lamellar architecture of AF, it is difficult to examine variation in mechanical properties by tensile testing as it can be undertaken like a tendon. We thus designed a bone-AF-bone specimen, to examine the differences in the mechanical properties before and after decellularization by tensile testing, using a testing protocol based on work by Woo et al. and Penn et al. [19,20]. Two parallel bone-AF-bone specimens were obtained from the same level disc and adjacent vertebrae with a width of 5 mm, depth of 8 mm, and bone stump length of approximately 15 mm (Figure 1A). Electronic vernier calipers were used to measure the exact length and cross-sectional area of the AF portion, and this value was used in calculations of the specimen properties. From the same disc, one sample was washed and stored at −20°C before testing, the other subjected to the decellularization procedure.

Bone-AF-bone biomechanics were assessed using a materials testing system (MTS, MTS Systems Corporation, MN, USA) in a uni-axial tensile test configuration. The distal 1 cm of each specimen was secured in a custom made grip (Figure 1B). The specimen was inspected throughout each test to ensure that no slippage occurred. At the beginning of each test, the specimen was preconditioned by running at 1 Hz at 1-mm displacement. Preconditioning was followed by monotonic tensile test-to-failure at a rate of 0.06 mm/s. Failure was defined as AF rupture or bone stump breakage. Load–displacement data were recorded at a frequency of 100 Hz by the MTS system. From the load–displacement curves, stiffness and Young’s modulus were obtained.

### Data analysis

All numerical data were analyzed using Microsoft Excel 2010. Means, standard deviations, and 95% confidence limits were calculated for each set of results. Data from fresh and decellularized specimens were compared using a paired Student’s *t*-test. In all tests, the threshold for statistical significance was *p* < 0.05.

## Results

### Histological analysis

**Figure 2:**
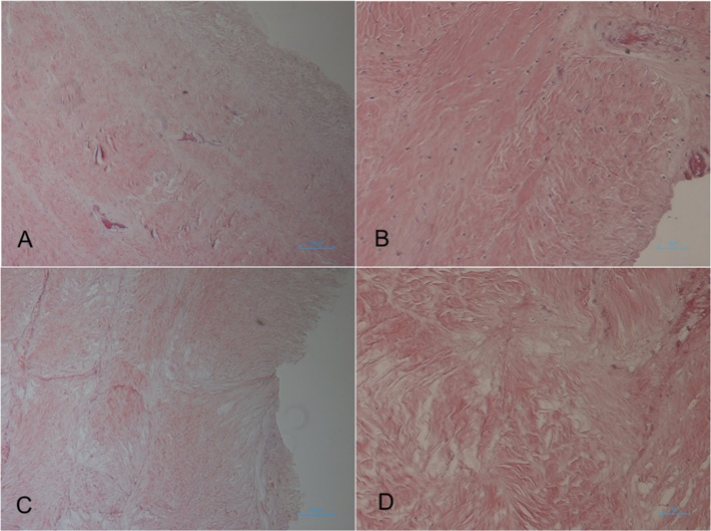
**Histology of anulus fibrosus (AF) with H & E stain.** Cellular material is clearly visible in the fresh-frozen AF **(A, B)** and rare to absent in the decellularized AF **(C, D)**. **(A)** Fresh AF, ×40 magnification; **(B)** Fresh AF, ×100 magnification; **(C)** Decellularized AF, ×40 magnification; **(D)** Decellularized AF, ×100 magnification.

In fresh AF tissue, H & E staining showed abundant cellular material, specifically nuclear material, to be embedded in the matrix (Figure 2A,B). In tissues subject to decellularization, rare cellular material was shown on H & E staining, as shown in Figure 2C,D.

**Figure 3:**
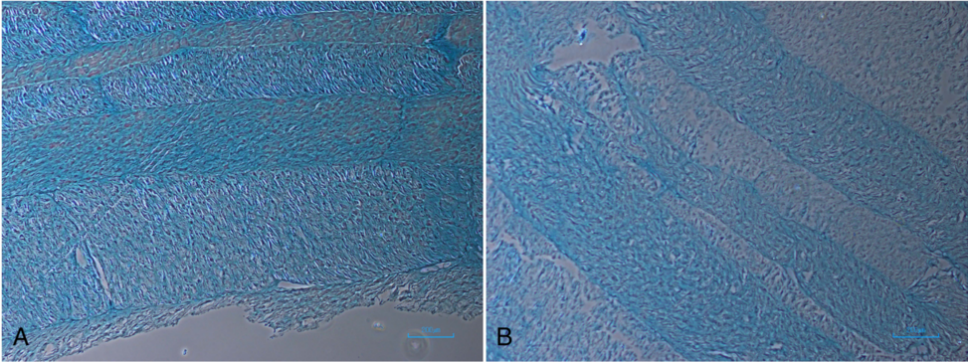
**Fresh and decellularized anulus fibrosus (AF) stained with alcian blue. (A)** Fresh AF stained with alcian blue, ×40 magnification. **(B)** Decellularized AF stained with alcian blue, ×40 magnification.

Alcian blue staining revealed the GAGs to be present over the entire fresh AF tissue in lamellar distribution (Figure 3A). In contrast, the decellularized matrix showed some qualitative loss of GAGs in all locations (Figure 3B).

**Figure 4:**
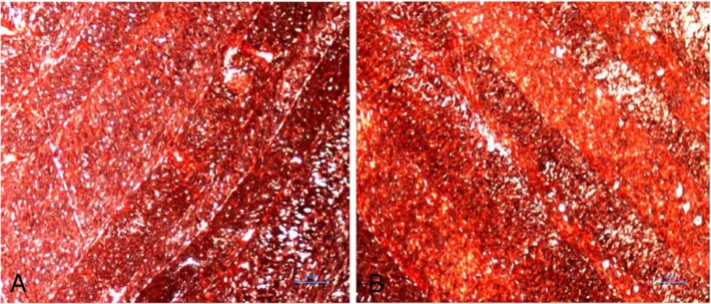
**Fresh and decellularized anulus fibrosus (AF) stained with sirius red. (A)** Fresh AF stained with sirius red, ×40 magnification. **(B)** Decellularized AF stained with sirius red, ×40 magnification.

Sirius red staining revealed plentiful collagen fibrils, which were visible in both fresh and decellularized AF without obvious changes in the morphology and distribution (Figure 4A,B).

#### SEM analysis

**Figure 5:**
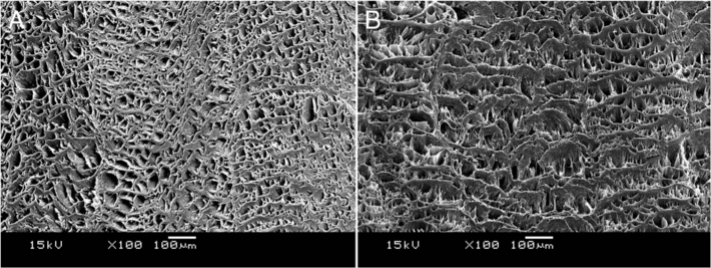
**SEM images of anulus fibrosus (AF). (A)** Image of fresh AF, presence of pore-like structure, ×100 magnification. **(B)** Image of decellularized AF, confirmed retention of pore-like structures on the AF surface, ×100 magnification.

SEM revealed the presence of small pore-like structures on both the fresh AF and decellularized AF surfaces, confirming retention of pore-like structures after decellularization treatment (Figure 5A,B).

### Biochemical assays

#### Quantification of GAGs

The result indicated loss of 15.9% GAGs following decellularization. However, there was no statistically significant difference between the sulfated sugar content of the fresh AF as compared to the decellularized AF (*p* > 0.05) (Table 1).

**Table 1:** **GAGs, hydroxyproline, and DNA contents of the fresh and decellularized anulus fibrosus (AF)**

	**Fresh AF (** ***n*** **= 6)**	**Decellularized AF (** ***n*** **= 6)**
GAGs (μg/mg)	13.90 ± 1.65	11.69 ± 1.74
Hydroxyproline (μg/mg)	98.65 ± 4.71	96.72 ± 3.33
DNA content (ng/mg)	31.58 ± 8.31	4.02 ± 1.56

Results given as mean ± SD.

#### Quantification of hydroxyproline

There was no significant difference in the hydroxyproline content of the fresh AF in comparison with decellularized AF (*p* > 0.05) (Table 1).

#### Quantification of DNA

DNA content of the decellularized AF scaffolds was significantly decreased by 86% when compared to the untreated AF (*p* < 0.01) (Table 1).

### Cytotoxicity assay

**Figure 6:**
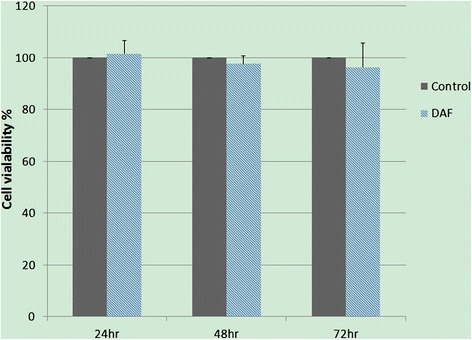
**Cytotoxicity studies of the control and decellularized anulus fibrosus (AF).** Data revealed no statistically significant difference of the decellularized AF in comparison with the negative control. *Control* negative control, *DAF* decellularized AF.

The *in vitro* toxicity studies of the control and decellularized AF were conducted by MTS assay with NIH3T3 cells. There was no statistically significant difference in *in vitro* cytotoxicity of the decellularized AF in comparison with the negative control (*p* > 0.05) (Figure 6).

### Tensile testing

From the load–displacement results of tensile testing, stiffness and Young’s modulus were obtained (Table 2). Tensile testing of the decellularized AF scaffolds showed a tendency of reduction of stiffness and Young’s modulus when compared to the untreated AF but that the difference was not statistically significant (*p* > 0.05). Specimens that failed at the grips were excluded from calculations.

**Table 2:** **Biomechanical properties of fresh and decellularized anulus fibrosus (AF)**

	**Fresh AF (** ***n*** **= 5)**	**Decellularized AF (** ***n*** **= 5)**
Stiffness (N/mm)	22.24 ± 5.07	19.95 ± 6.53
Young’s modulus (MPa)	62.58 ± 9.19	60.7 ± 14.86

Results given as mean ± SD.

## Discussion

We have reviewed various decellularization protocols with different chemical, physical and enzymatic methods [21-25]. At present, complete decellularization of tissue will require a combination of physical, chemical, and enzymatic approaches [6]. Compared to a single freeze-thaw treatment, the repeated freeze-thaw of tissues is known to produce most cell destruction and has been used frequently for decellularization of tendinous and ligamentous tissue [23,26]. Following freezing, a subsequent incubation in 0.1% (*w*/*v*) SDS and nucleases (RNase and DNase) is used to solubilize the cellular remains. Finally, extensive washing in PBS will further remove cell remnants and all residual chemicals. Results of H & E staining showed that decellularization was achieved through modified decellularization protocols. To further verify the efficiency of cell removal, DNA content was examined by quantitative spectrophotometry. Results revealed 86% reduction in DNA content in the decellularized AF scaffold when compared to the non-treated AF tissue.

Following decellularization, it was important to determine the effects on the biochemical and biomechanical properties of the decellularized scaffold, while preliminarily evaluating its biocompatibility. GAGs and collagen are extracellular matrix components, important for maintaining the biologic and mechanical properties of AFs. Alcian blue staining revealed that the GAGs were still present after decellularization, but there seems to be partial qualitative loss. Loss of GAGs was further verified by sulfated glycosaminoglycan assay, the results indicating that 15.9% GAGs were removed during the processing steps, although there was no statistically significant difference when compared to the non-treated AF tissue. Collagen is the major stress-bearing component of fibrous tissue, playing an important role in the tensile properties of the AF tissue. Sirius red staining revealed that collagen fibrils abound in both fresh and decellularized AF without gross evidence of damage and distribution. Hydroxyproline is one of the main elements of collagen and can thus be used to determine the levels of collagen. Further investigation revealed that there was no significant change in hydroxyproline content after decellularization.

Although the decellularization methods described above include chemicals and enzymes, which are utilized for their inherent abilities to destroy cells, if the chemicals remain within the tissue after treatment, they may be toxic to host cells while implanting *in vivo*. In an effort to remove any remaining chemical and enzymatic reagents, extensive washing in PBS at the end of the protocols was applied. Simple *in vitro* cytotoxicity tests were performed to examine the biocompatibility of decellularized AF scaffolds, which indicated that any residual, potentially cytotoxic reagents had been adequately removed by the wash procedure.

A successful strategy for AF repair requires a clear understanding of the functional biomechanics of the disc. The NP is highly pressurized and the AF prevents radial disc bulge by generating large hoop stresses. Therefore, AF repair materials need to withstand the high tensile hoop stresses generated from NP pressurization and tensile stresses resulting from spinal motion [4]. Tensile testing of the decellularized AF demonstrated no significant difference in stiffness or Young’s modulus, further indicating to the satisfactory preservation of biomechanics in comparison to the native tissue. These results demonstrate that the decellularization protocol preserved desirable components of the AF matrix, which suggests that acellular AF would be a potential scaffold for clinical utility.

Although the above methods provide important information regarding the effectiveness of the decellularization methods, consideration must be given to the potential immune response of small amounts of nuclear material or cytoplasmic debris within the remaining scaffold materials. It has been found that the mammalian cell-surface xenoantigen, α-Gal epitopes (Galα1,3-Galβ1-4GlcNAc-R), can trigger a hyperacute rejection after transplant of living animal tissue to humans. Various efforts had been carried out to eliminate the α-Gal epitopes from xenograft to reduce the immune response. The α-Gal epitopes have been found in small intestinal submucosa-extracellular matrix (SIS-ECM), a biomaterial consisting of porcine small intestinal submucosa [27]. To simulate the possible implications in human implantation, the role of α-Gal epitope in the host immune response to SIS-ECM has been investigated in previous studies. These demonstrated the presence of anti-Gal antibody delays but that the immunomodulatory effect of Gal epitope does not adversely affect *in vivo* remodeling of xenogeneic extracellular matrix (ECM) [28,29]. Most commercially available biologic scaffold materials also contain trace amounts of remnant DNA, which is typically present as small fragments and logically subject to degradation *via* enzymatic breakdown [7,30]. That the remaining DNA fragments and α-Gal epitopes are capable of stimulating an immune reaction is well known, but it is possible that a threshold amount of material is required to adversely affect the remodeling response [31]. It is unlikely that any combination of methods will remove 100% of all cell components from a tissue or organ. However, it seems apparent that methods which remove most or all of the visible cellular material result in biologic scaffold materials that are safe for implantation [6].

## Conclusion

In this work, we demonstrate effective methods of decellularizing porcine AF tissue while retaining the biologic composition and ultrastructure of the ECM. Additionally, we describe that biomechanical properties could be successfully preserved in a decellularized AF scaffold. Although tissue engineering AF tissue remains a challenge, our data suggest that this scaffold is biocompatible and a potential candidate for clinical application. Future work will optimize cell seeding and test the decellularized AF scaffold in a functional large animal model of disc repair. This will establish the *in vivo* regenerative capacity of the porcine AF scaffold over the longer term. Alternative surgical options with novel strategies towards anular repair would significantly improve the chances for the long-term benefit of discectomy procedures.
